# Artifacts in cardiac T1 and T2 mapping techniques—Influence on reliable quantification

**DOI:** 10.1016/j.jocmr.2025.101934

**Published:** 2025-07-26

**Authors:** Maximilian Fenski, Jan Gröschel, Peter Gatehouse, Christoph Kolbitsch, Jeanette Schulz-Menger

**Affiliations:** a Charité – Universitätsmedizin Berlin, corporate member of Freie Universität Berlin and Humboldt-Universität zu Berlin, Experimental and Clinical Research Center, Berlin, Germany; b Division of Cardiovascular Medicine, Radcliffe Department of Medicine, University of Oxford, Oxford, United Kingdom; c Physikalisch-Technische Bundesanstalt (PTB), Berlin, Germany

**Keywords:** Cardiovascular magnetic resonance, T1 mapping, T2 mapping, Magnetic resonance imaging artifacts, Image quality assurance

## Abstract

Cardiac T1 and T2 mapping techniques are well-established methods for obtaining quantitative pixelwise representations of myocardial tissue properties. Mapping images are commonly evaluated quantitatively, and their resulting values play a crucial role in diagnosis and therapeutic decision-making in various cardiac pathologies. Despite the validated effectiveness of these techniques, both methodological and patient-specific confounders must be considered when applying them in clinical and research settings. Artifacts—erroneous features within the magnetic resonance image—can be misinterpreted as true anatomical structures or pathologies, potentially confounding quantitative analyses, conducted by both human readers and artificial intelligence algorithms. Artifacts can arise from sources such as patient motion, metal objects, hardware constraints, patient-specific scanner adjustments (e.g., flip-angle calibration), and processing errors, particularly within the complex environment of cardiac imaging. While artifact sources in other cardiovascular magnetic resonance sequences are well-documented, cardiac parametric mapping presents unique challenges due to its distinct image generation and quantitative assessment. This article provides an overview of artifacts encountered in cardiac T1 and T2 mapping, along with a concise explanation of their origins, aiming to raise awareness of their potential impact on clinical decision-making. Future developments, including sequences designed to mitigate mapping artifacts, are also briefly discussed. A strong interaction between scientists and clinicians is needed to overcome these challenges and maintain the reliability of quantification results.

## Graphical abstract

**Figure:**
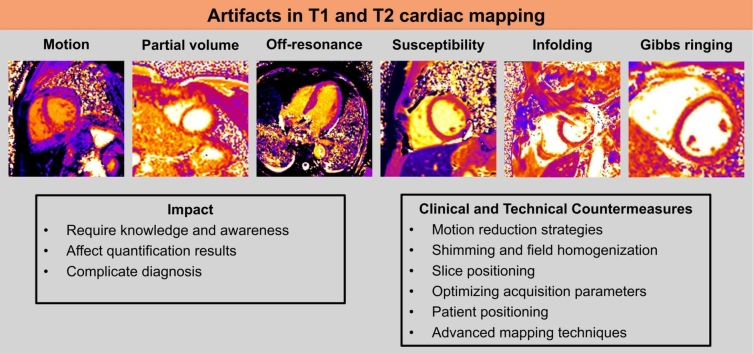

## 1 Introduction

T1 and T2 parametric mapping techniques are validated and reliable methods for generating detailed pixelwise maps of the heart, where each pixel represents an estimate of the T1 or T2 value of the underlying tissue (1–7)(1–7) [1], [2], [3]. These parametric maps allow for the quantitative analysis of T1 or T2 deviations from normal values, aiding in the detection of various myocardial pathologies and their treatment response [4], [5], [6], [7], [8], [9], [10]. The technical principles and clinical applications of T1 and T2 mapping in the heart have been well-documented [11], [12], [13], [14], [15], [16], [17] and continue to evolve. When applying mapping techniques in clinical practice or research, it is crucial to consider both methodological and patient-specific confounders [15]. Additionally, clinicians involved in image analyses should have a basic technical understanding of the acquisition process and be capable of assessing the quality of the final map as well as the individual source images used to create it. This includes the ability to identify artifacts and other sources of errors.

In magnetic resonance imaging (MRI), artifacts are common and refer to any (erroneous) feature in the image that does not represent the true anatomy or pathology of the subject [18]. These errors can arise from various sources, including patient or organ movement, hardware limitations, software processing, or external factors and objects. In cardiovascular magnetic resonance (CMR), the anatomical complexity of the chest, combined with cardiac and respiratory motion, and blood flow, and distortion of the main magnetic field are significant sources of artifacts. Artifacts can severely degrade image quality, complicating diagnosis and potentially leading to incorrect interpretations. However, artifacts can also be subtle, not obvious to the eye, yet affect the estimated T1 or T2 values. Considerable effort has been made to describe artifacts in basic CMR sequences [18], [19], [20], [21], [22], [23]. However, the unique sequence architecture in cardiac parametric mapping, where the final map is generated from multiple source images, coupled with quantitative non-linear evaluation and the increasing use of artificial intelligence (AI)-supported analysis methods, requires specialized knowledge and awareness of artifacts in cardiac mapping procedures. In addition, decisions must be made as to whether only parts of a mapping image are to be excluded from evaluation or whether the entire image is to be discarded. This article is intended for a clinical reader, aiming to highlight the occurrence of artifacts in cardiac T1 and T2 mapping images and to assist in distinguishing artifacts from true pathologies.

The article begins with a brief overview of the basic T1 and T2 mapping acquisition concepts, followed by examples of common artifacts, with a concise explanation of the underlying mechanisms whenever possible. This overview focuses on sequences that are available on many scanners but will also give an outlook on sequences that have been developed to overcome commonly observed artifacts in cardiac mapping sequences.

## 2 Informed consent and patient details

The images included in this review were retrospectively obtained from clinical routine scans or dedicated research studies. Their retrospective use was approved by the local ethics committee of Charité – Universitätsmedizin Berlin (study ID: EA 1253 21) and complies with the Declaration of Helsinki.

## 3 Technical principles of T1 and T2 mapping

### 3.1 T1 mapping principles

**Fig. 1:**
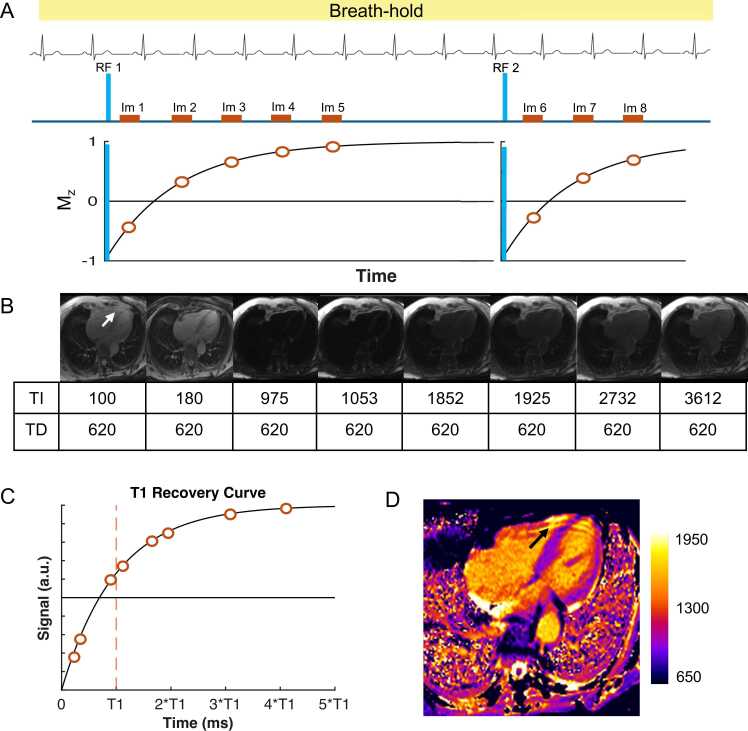
T1 Mapping principle for a commonly used native T1 setup (“MOLLI 5(3)3”). (A) Following an 180° inversion pulse (RF 1), five single-shot images (i.e., source images, “Im”) are intermittently acquired during diastole, using a fixed trigger delay (TD). The first image is acquired at a fixed inversion time (TI) of 100 ms, while subsequent TI values increase based on the R-R interval length. The first set of image acquisitions is followed by an Mz recovery period of three R-R intervals. Some scanners run “dummy” gradients (without any RF pulses) in the recovery gaps to avoid patient breathing. Following a second inversion pulse (RF 2), three more source images are acquired with increased TI values, sampling additional points along the Mz recovery curve. The readout module (“Im”) commonly uses a single-shot steady-state free precession technique. (B) All source images are acquired in a single breath-hold and ordered according to their TI. Non-rigid registration techniques are often applied to correct for residual in-plane motion between source images, which can improve the robustness of the method. Some sequences provide magnitude- and phase-sensitive inversion recovery reconstructions (C). Signal intensities across the source images are fitted to an exponential recovery curve to estimate the T1 time for each specific pixel. (D) The final map is a pixelwise representation of the estimated T1 values. Artifacts, such as the one shown in source images (white arrow in “B”), may also appear in the final map (black arrow in “D”). However, artifacts can sometimes be easily overlooked, as will be demonstrated in the subsequent sections. Note that the impact of the single-shot images on the Mz recovery curve is not shown here. This impact affects the look-locker set of points after one inversion pulse. The readout sequence distorts the fitted T1 (known as T1*) and requires correction known as look-locker correction. Note that the band affecting the apex resembles that described in Fig. 11. *RF* radiofrequency. *MOLLI* modified Look-Locker inversion recovery

T1 (spin-lattice) relaxation time is a time constant of the longitudinal magnetization. It refers to the period needed for the longitudinal magnetization to recover approximately 63% of its initial equilibrium value (known as M_0_) after a 90° saturation pulse. Native myocardial T1 values are shortened by fat and iron and are prolonged by tissue-free water content and expansion of the extracellular space [15]. The general principle of T1 mapping is to obtain several images—often called “raw” or “input” (to map calculation) or “source” images—at different time points after a preparation pulse (e.g., inversion pulse or saturation pulse) and sort them in order of increasing time after the preparation pulse [24]. The signal in each pixel is fitted to a model describing the T1-dependent behavior of the longitudinal magnetization [14]. T1 times can then be determined for each pixel location, and pixel intensities in the final mapping image (i.e., “T1 map”) correspond to the fitted T1 values. All routinely used T1 mapping methods rely on a combination of magnetization preparations and T1-weighted image acquisitions over multiple cardiac cycles, typically during a single breath-hold [24], [25], [26]. Longitudinal magnetization is prepared using either inversion or saturation radiofrequency (RF) pulses [27]. T1-weighted images are then acquired using single-shot techniques, where all the data for each image is acquired in a single heartbeat. Image acquisition is commonly performed using balanced steady-state free precession (bSSFP) or spoiled gradient echo (sGRE) readout sequences. The most widely used T1 mapping techniques are the modified look-locker inversion recovery (MOLLI) sequence [1], its variants (e.g., shortened modified look-locker inversion recovery [ShMOLLI] [25]), and the saturation recovery-based saturation recovery single-shot acquisition sequence (SASHA) [26]. Fig. 1 illustrates the MOLLI 5(3)3 acquisition scheme, which is typically applied to native T1 and performed in a breath-hold over 11 heartbeats.

### 3.2 T1 fitting and Look‐Locker correction

For each pixel, the T1-dependent behavior of longitudinal magnetization recovery following a magnetization preparation can be estimated by fitting the signal to a three-parameter model: *S*(ti) = *A* − *B* exp(−ti/T1*)[24]. However, due to the influence of repeated RF pulses used to acquire the individual source images, the measured signal deviates from the ideal inversion recovery curve. Instead, it follows a modified, faster recovery curve, resulting in an apparent relaxation time referred to as T1*. As T1* underestimates the true T1, a correction—commonly the look-locker correction—is applied with T1 = T1*(*B*/*A* − 1) to derive the actual T1 from T1*. Importantly, the look-locker method assumes perfect inversion efficiency. Imperfect inversion, caused by transverse relaxation during the inversion pulse, as well as B0 or B1+ field inhomogeneities, may not produce obvious artifacts in the source images but can result in underestimation of T1 values [28], [29]. Some vendors apply an additional correction factor to account for transverse relaxation during the inversion pulse.

### 3.3 T2 mapping principles

**Fig. 2:**
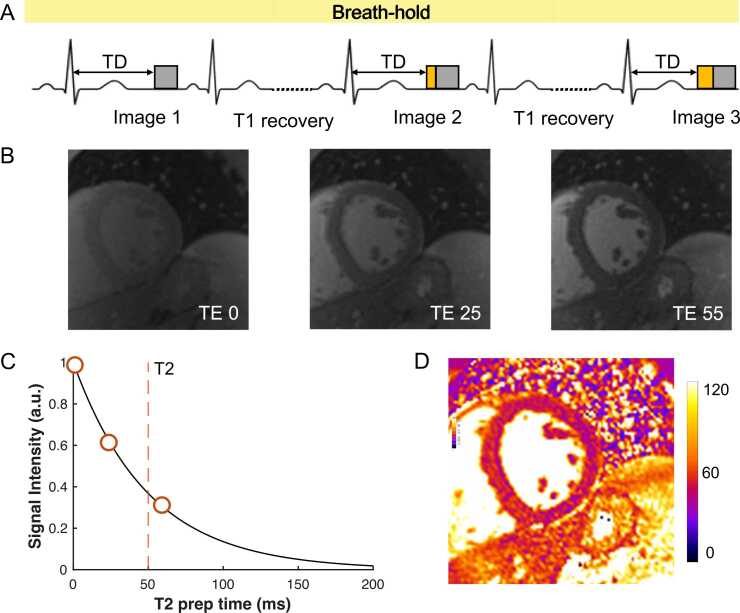
T2 Mapping principle. (A) A commonly applied T2 mapping acquisition scheme uses a T2-preparation model (yellow box) to induce T2-weighting, followed by a single-shot readout (gray box, commonly bSSFP or spoiled gradient echo approaches) to obtain a source image. Typically, no T2-preparation is applied to the first image as this acts as the initial value of the T2 signal decay curve. Parameters are selected to keep total acquisition within a breath-hold duration, and each T2-prepared image is captured in a single heartbeat, with a recovery period (e.g., 3 R-R intervals or 3 seconds) between acquisitions to allow T1 magnetization recovery. (B) Three source images are acquired at varying T2 sensitivities (here, echo times TE1 = 0 ms, TE2 = 25 ms, TE3 = 55 ms) and spatially co-registered. (C) Signal intensities are fitted to a mono-exponential decay model and used to (D) create the final quantitative parametric map, often color-coded for visualization. *bSSFP* balanced steady-state free precession, *TD* trigger delay, *TE* echo time

T2 (spin-spin) relaxation time refers to the period needed for spin-echo rephased magnetization to decay to approximately 37% of its initial value following a 90° excitation pulse. Elevated T2 times can indicate increased myocardial water content or alterations in water distribution without a net increase in water content [3], [30]. T2 mapping is commonly used to detect conditions such as acute myocarditis [31], myocardial inflammation in chronic systemic inflammatory diseases [32], [33], [34], and acute myocardial infarction [8]. The fundamental principle of T2 mapping involves acquiring several single-shot images after different T2 preparations and fitting the resulting signal in each pixel to a model that describes the T2-dependent behavior of transverse magnetization. Pixel intensities in the final T2 map correspond to the fitted T2 values. The most commonly used acquisition technique is a T2-preparation module with varying echo times (e.g., 0, 24, 55 ms) followed by a steady-state free precession or sGRE readout sequence, to obtain the source images [2], [35], [36]. Fig. 2 illustrates the acquisition scheme for a T2-prepared mapping sequence. T2 mapping can alternatively employ multi-echo fast spin echo [37] or gradient spin echo [38] techniques giving the finest spatial resolution, reviewed in O'Brien et al.[16] usually by segmented k-space raw data acquisitions; as for T2* above, this may cause other types of artifacts than the single-shot based mapping methods mainly covered in this review.

### 3.4 T2* mapping

T2* relaxation time refers to the period needed for transverse magnetization (without spin-echo rephasing) to decay to approximately 37% of its initial value following an RF excitation pulse. T2* is a relaxation parameter that includes local magnetic field inhomogeneities (T2′); these inhomogeneities increase with iron deposition [39], shortening T2*. T2* values are estimated by acquiring multiple images at increasing echo times (∼2–20 ms) and fitting the signal decay on a pixelwise basis to an exponential curve [40]. However, as T2* mapping normally uses segmented k-space raw data acquisition, the encountered artifacts may differ from T1 and T2 mapping, which is usually single-shot imaging. For detailed discussion on technical factors influencing T2* mapping, readers are referred to previous publications [41], [42].

## 4 Quality Inspection

Two primary sources of error can degrade the accuracy and precision of final T1 and T2 map values in clinical practice: misalignment between source images and artifacts present in one or more source images. As a result, raw and motion-corrected source images, the final map, and available quality control maps (e.g., R² or error maps) should be systematically inspected after acquisition [15]. Most vendor-provided error maps offer a pixelwise visualization quantifying the fitting error, indicating how well the input pixel values conform to the T1 or T2 equation. Regions with high error values may suggest residual motion or artifacts on source images, warranting closer inspection to identify the underlying cause (Fig. 4C). Additionally, all acquired images should be inspected for consistent slice positioning, cardiac phase synchronization, the absence of mis-triggered or skipped heartbeats during data acquisition and to confirm the success of motion correction (MOCO) (15). Recognizing through-plane motion is particularly important when using MOLLI sequences, as it can modulate the effects of image readouts on the magnetization history assumed for Look‐Locker correction. Notably, commonly used retrospective MOCO methods can only address in-plane motion to a limited extent and cannot correct for through-plane motion. Since identifying the specific source of patient-related artifacts retrospectively is often difficult or even impossible, effective patient communication and real-time assessment of the electrocardiogram (ECG), the trigger quality, and respiratory position (on some systems, with limitations) are essential during image acquisition. The following sections will describe commonly encountered artifacts. Future developments may enable fully automatic quality control, reducing the need for labor-intensive and time-consuming manual assessment [43].

**Fig. 3:**
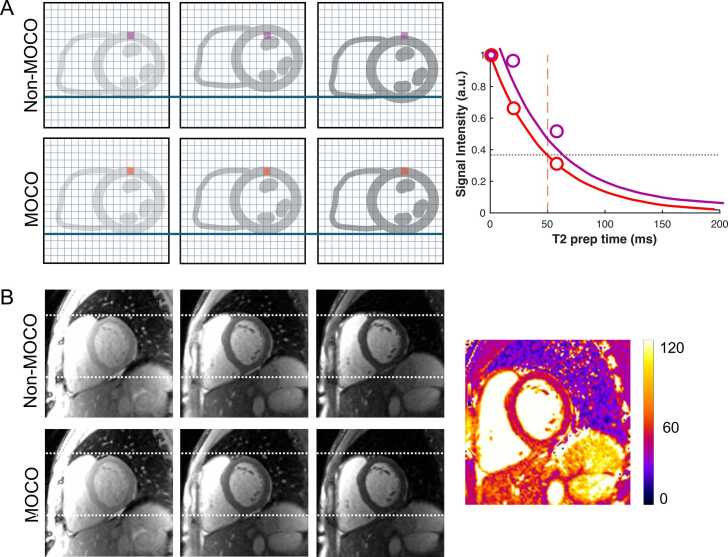
Importance of anatomical alignment across source images for reliable map fitting. (A) Three source images with different T2 preparations are shown, with an overlayed grid representing individual pixels. Without motion correction (“Non-MOCO”), residual motion between acquisitions caused misalignment of the heart, resulting in different tissues being represented within the same pixel (illustrated in purple). This leads to an erroneous T2 recovery curve fit and incorrect T2 measurements. Following motion correction, consistent anatomical alignment across source images was achieved, allowing accurate T2 curve fitting and accurate measurements (illustrated in red). (B) Anatomical misalignment is evident in the source images (e.g., the white dashed line across the left ventricular myocardium in image three) before motion correction. Following motion correction, proper alignment was restored, enabling the generation of a reliable final T2 map. *MOCO* motion correction

**Fig. 4:**
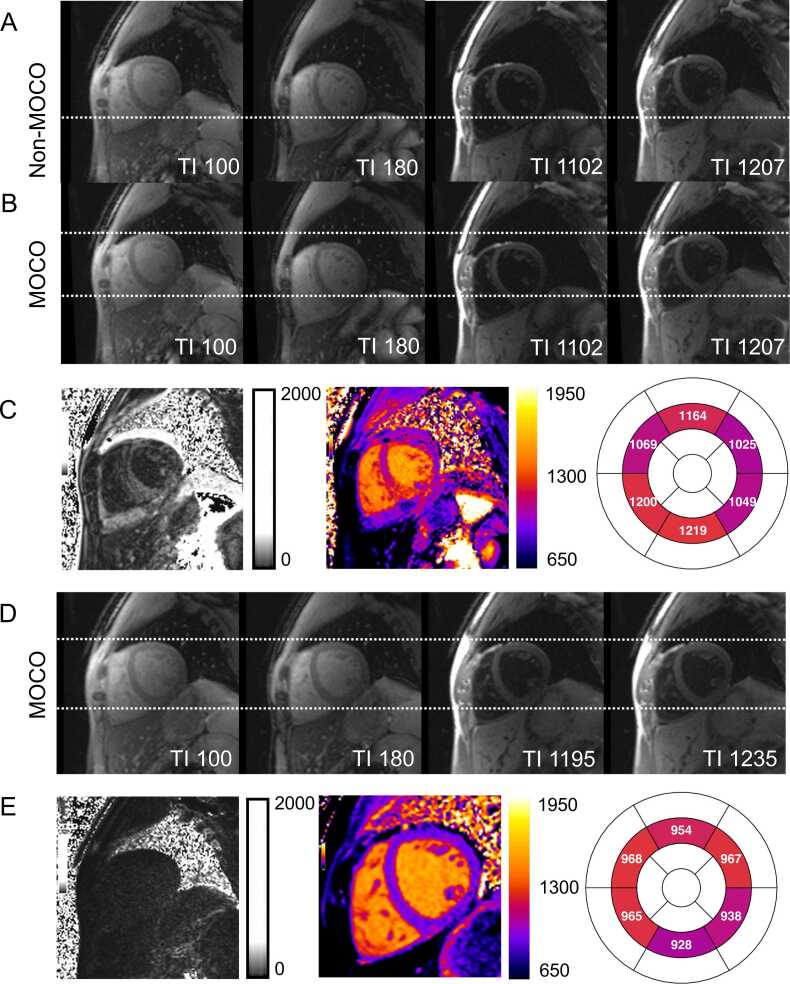
Failed motion correction. (A) Residual motion between source images is evident, as indicated by the varying position of the myocardium relative to the dashed white line. Residual motion is most likely caused by different breath-hold positions, indicated by different lung areas on source images TI = 100 and TI = 180 ms (the purple area on both images is identical). (B) The applied non-rigid motion correction algorithm [44] failed to properly align the anatomical structures across source images. (C) As a result, the corresponding error map reveals high uncertainty in the fitted myocardial T1 values and adjacent structures (white = higher uncertainty, black = lower uncertainty). Consequently, the final T1 map displays erroneously elevated T1 values outside the normal range (T1 2SD normal range: 905–1037 ms at 1.5T). (D) After reacquiring the data at the same slice position with an identical breath-hold position, motion correction was successfully applied. (E) The new error map demonstrates low uncertainty in the myocardial T1 fit, and the final T1 map presents well-defined myocardial boundaries with T1 values within the normal range

## 5 Motion artifacts

Reliable T1 or T2 mapping requires consistent anatomical alignment across all source images [45]. Residual motion during or between image acquisitions can result in variable positioning of the myocardium across source images, which can severely degrade mapping quality and reliability. Motion may arise from respiration, patient movement, cardiac contraction, or cardiac movement within the pericardial cavity. Anatomical misalignment can cause pixel values to be derived from different spatial locations, rather than accurately reflecting the T1 or T2 properties of a single anatomical site. Importantly, motion during acquisition and motion between acquisitions give rise to different types of artifacts.

### 5.1 Minimizing and correcting motion

Cardiac motion is typically minimized by acquiring source images at the most quiescent phase of the heart cycle, i.e., either at end-diastole or mid-systole, using an acquisition window of ∼175–250 ms [17]. Respiratory motion is minimized by acquiring all source images within one breath-hold or, less commonly, using diaphragm navigators or other respiratory gating techniques. However, residual inter-image misalignment due to respiration, arrhythmia, or variability in cardiac contraction can still degrade the reliability of the final map.

To address this, non-rigid MOCO algorithms are applied before pixel-by-pixel fitting [46], [47]. These algorithms align anatomical structures across source images, primarily correcting for respiratory motion. However, their efficacy is limited in cases of cardiac motion due to arrhythmias, premature ventricular contractions, or mistriggering, which can lead to residual anatomical misalignment or deformation [48]. Figs. 3 and 4 illustrate the importance of precise anatomical alignment across source images and the consequences of failed MOCO.

### 5.2 Contrast variability and image registration methods

A key challenge in MOCO arises from the varying contrast of source images. As mentioned above, MOCO is carried out by first estimating the motion (i.e., deformation) between the individual source images. Commonly, non-rigid image registration algorithms are used to capture the complex motion of the heart accurately. If the intensity of, and the contrast between, anatomical features change between source images, a motion estimation algorithm may mistake these contrast changes for motion and incorrectly apply transformations during the MOCO step, leading to further artifacts. A wide range of approaches to address this challenge have been proposed [44], [46], [47], [49], [50], [51], [52]. One commonly used strategy is to incorporate the signal model to predict intensity changes caused by, for example, inversion or T2-preparation pulses. This helps to distinguish between signal changes due to motion and those due to the contrast mechanisms required for quantitative mapping, thereby enabling more robust motion estimation.

### 5.3 Cardiac and respiratory motion

**Fig. 5:**
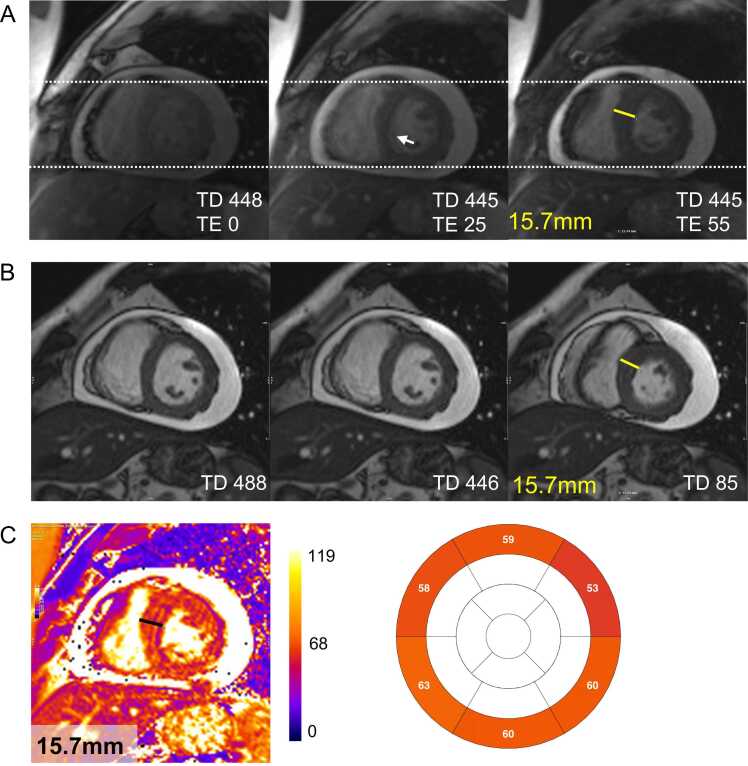
Mistriggering and residual cardiac motion. A patient with arrhythmias and a large circumferential pericardial effusion presented with heart “swinging” within the pericardial cavity. (A) T2-weighted source images erroneously acquired at different cardiac phases demonstrate noticeable variations in septal wall thickness. The trigger delay (TD) values extracted from the DICOM data may be misleading in cases of extreme arrhythmia. The white arrow in the second image highlights residual cardiac motion, resulting in blurred myocardial borders. (B) For comparison, short-axis cine images acquired at the same slice location can help estimate the approximate cardiac phase in which the individual source images were obtained. (C) Myocardial T2 values in the final map likely represent a mixture of different tissues, such as blood, myocardium, and pericardial effusion. The map should not be considered for clinical interpretation. *DICOM* digital imaging and communications in medicine, *TE* echo time

**Fig. 6:**
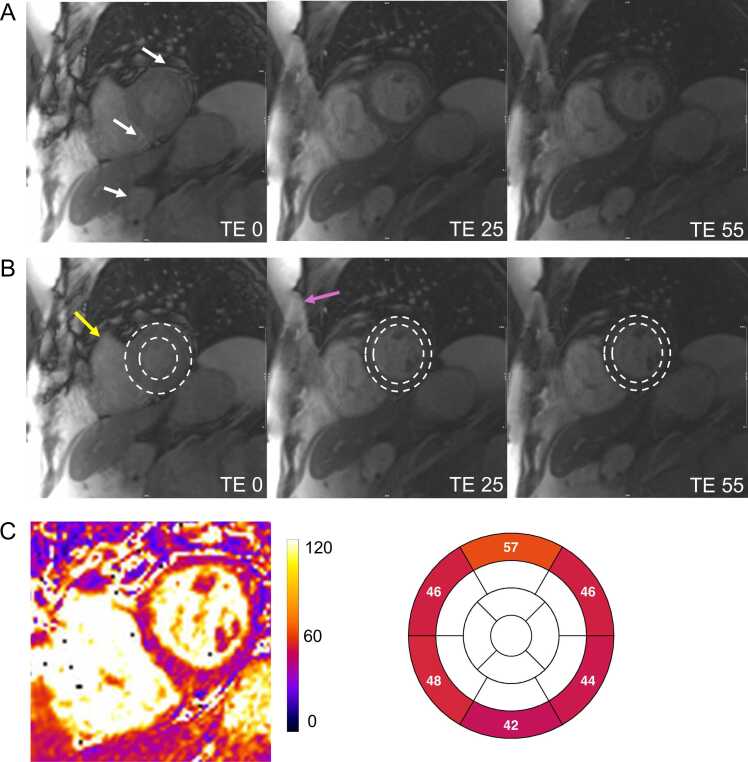
Residual in-plane and through-plane motion, possible parallel imaging, and infolding artifacts in a patient with frequent arrhythmias and impaired breath-holding. (A, B) Mistriggering during acquisition resulted in the first source image being captured during a more systolic phase, as indicated by the increased left ventricular wall thickness compared across images (B, dashed line). Through-plane motion is further highlighted by the presence of the right ventricular outflow tract (B, yellow arrow) and pulmonary valve, which are absent in the diastolic phase images. Additionally, an infolding artifact (pink arrow) is observed. White arrows point to another artifact, likely a parallel imaging motion artifact, due to respiratory motion. (C) For parallel imaging, GRAPPA with an acceleration factor of 2 and separate coil information measurements was applied. As a consequence, the final T2 map erroneously displays pathologically elevated T2 values in the basal anterior segment and pathologically low T2 values in the basal inferior segment. However, the presence of the described artifacts compromises the accuracy of the resulting T2 map, rendering it unsuitable for reliable analysis or clinical decision-making. Note that the parallel imaging ghost does not appear exactly at half the distance from the bright anterior chest wall due to image cropping. *GRAPPA* generalized autocalibrating partially parallel acquisition, *TE* echo time

Residual cardiac motion during the acquisition window can cause blurred myocardial borders (Fig. 5A). Inconsistent triggering or arrhythmias may result in acquisition during different cardiac phases, leading to variations in left ventricular wall thickness or changes in the heart’s position relative to surrounding structures (Figs. 5 and 6).

**Fig. 7:**
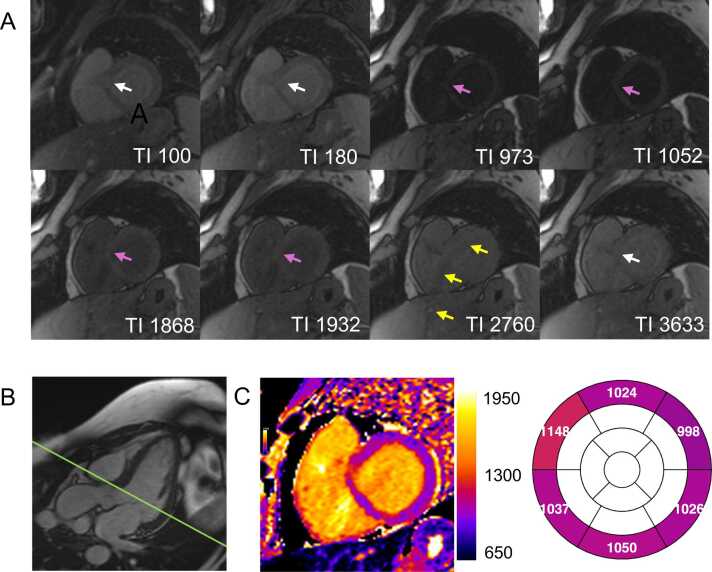
Through-plane motion and partial volume due to slice positioning close to the left ventricular outflow tract (LVOT). (A) Some of the source images show partial coverage of the LVOT (white arrows). Other source images were acquired at a slightly more mid-ventricular position, capturing the LVOT to a lesser extent (pink arrows), reflecting residual through-plane motion between source image acquisitions. Additionally, a parallel imaging motion artifact (yellow arrows) is visible. For parallel imaging, GRAPPA with an acceleration factor of 2 and separate coil information measurements was applied. (B) LVOT view cine imaging confirms the short-axis slice positioning near the LVOT (green line). (C) The final map demonstrates elevated T1 values in the anteroseptal wall, reflecting through-plane motion and partial volume effects from the mixture of blood and myocardium. Motion correction approaches, such as coregistration, are only capable of correcting for in-plane motion and cannot compensate for through-plane motion. Note that the parallel imaging ghost does not appear exactly at half the distance from the bright anterior chest wall due to image cropping. *GRAPPA* generalized autocalibrating partially parallel acquisition, *TI* inversion time

Sometimes, non-rigid MOCO can distort the very different cardiac shape in the misgated source image to match the reference image. Through-plane motion or discrete patient movement can cause anatomical features, such as the left ventricular outflow tract (LVOT), right ventricular outflow tract, or papillary muscles, to appear or disappear across source images (Figs. 6B and 7). In patients with arrhythmias, systolic acquisition may yield more consistent alignment [53], [54], [55]. ShMOLLI-derived T1 may be particularly susceptible to mistriggering, as ShMOLLI employs conditional data analysis that selectively discards source images based on the estimated T1 value and the average length of the R-R interval during the breath-hold. Emerging self-gated approaches that use continuous acquisition with retrospective data sorting offer the potential for free-breathing, ECG-independent mapping [56], [57].

### 5.4 Respiratory motion and parallel imaging artifacts

Poor breath-holding can significantly impair the quality of parametric maps. Respiratory motion can occur both between and during source image acquisition, often manifesting as diaphragmatic motion or blurring of tissues and borders that are mobile with respiration. Since single-shot techniques are used, the speed of respiratory motion is unlikely to vary sharply enough during data acquisition to cause other typical motion artifacts, such as ghosting, which is more commonly seen with some strategies (interleaved ky) for segmented k-space breath-hold acquisition. However, respiratory misregistration can introduce parallel imaging (PI) artifacts into the source images. PI is a widely used MRI technique that utilizes the spatial sensitivity information of multiple receiver coils to reduce phase-encoding steps and thereby accelerate image acquisition [58]. In PI, the sensitivity of each coil toward a tissue is highest near its physical location, providing additional spatial information [59] known as the coil’s sensitivity information. The acquired k-space data are then reconstructed using algorithms that utilize the coil sensitivity information. The two most common PI approaches are sensitivity encoding (SENSE) [60] and generalized autocalibrating partially parallel acquisitions (GRAPPA) [61]. For SENSE, coil sensitivity maps are estimated in image space from the coil sensitivity information, and for GRAPPA, a k-space–based kernel is estimated from the coil sensitivity information. In both cases, the coil sensitivity information usually comprises fully sampled low-resolution k-space data, which is either acquired before, during, or after mapping acquisition. For accurate image reconstructions, the coil sensitivity information must be consistent with the main single-shot data used for mapping [58]. If respiratory motion causes inconsistencies between the coil sensitivity information and the image data, PI artifacts can appear. These artifacts occur along the phase-encoding direction at locations precisely related to the degree of PI undersampling. In typical cardiac mapping, where an acceleration factor of 2 is used, PI artifacts often appear exactly half the phase-encoding field of view (FOV) away from bright tissue regions that have shifted due to motion (Figs. 6 and 7).

The simplest solution to PI-related artifacts is to repeat the scan after reminding the patient to follow breath-hold instructions. However, some patients may be unable to sustain an adequate breath-hold for the required duration. While some mapping sequences acquire coil sensitivity information separately, other mapping setups acquire coil profiles during each single-shot acquisition, which should almost eliminate PI ghosting from respiratory misregistration and improve the signal-to-noise ratio, albeit at the cost of slightly longer single-shot durations. Recently, navigator- or pilot-tone–based techniques have been proposed to correct both in-plane and through-plane motion, potentially enabling reliable free-breathing T1 and T2 mapping [62], [63]. However, in free-breathing mapping methods, ensuring a sufficiently long magnetization recovery time before acquiring the first source image is critical. If magnetization (M₀) does not fully recover before the first acquisition, this can introduce bias and distort the calculated T1 and T2 values. In standard breath-hold acquisitions, this recovery period naturally occurs between breath-holds and is enforced by patient instructions.

## 6 Partial volume effects

**Fig. 8:**
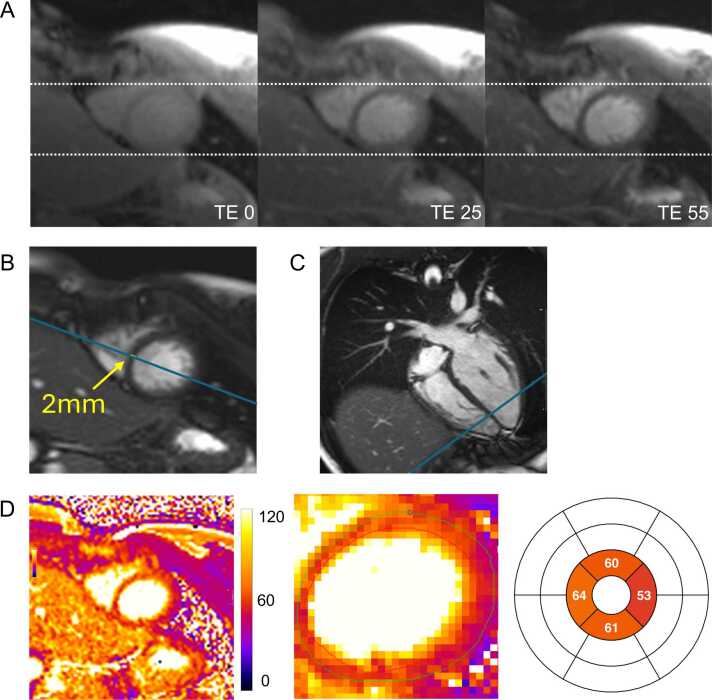
Partial volume effect due to thin septal wall. (A) T2-weighted source images acquired in end-diastole (TD 802 ms) show no artifacts and good anatomical alignment. (B) bSSFP short-axis cine imaging, also acquired in end-diastole (TD 798 ms), confirms low apical septal wall thickness. Blue lines indicate the reference positions of the four-chamber view (“B”) and the corresponding short-axis view (“C”). (D) The final T2 map (D, first image) demonstrates severely elevated T2 values in three of the four apical segments (institutional T2 upper limit of normal at 1.5T: 52 ms). The in-plane resolution of the T2prep-bSSFP mapping sequence used is 1.6 × 1.6 mm. Therefore, the true myocardial wall thickness is represented by approximately 1 voxel (D, second image, non-interpolated view). The remaining adjacent voxels likely represent a mixture of myocardium and blood due to partial volume effect, resulting in artificially elevated T2 values. *bSSFP* balanced steady-state free precession, *TD* trigger delay, *TE* echo time

In MRI, the final image is composed of thousands of voxels, each representing a small volume of the examined object. The signal intensity in a given voxel depends on the magnetic properties of the corresponding anatomical tissue and the specific sequence used to acquire the image [64]. When a voxel contains a mixture of different tissue types, i.e., spin populations with different intrinsic T1 value, such as fat and muscle, its signal represents a weighted combination of their respective relaxation properties. However, standard fitting models used to generate T1 or T2 maps assume a single-exponential recovery or decay, which may not accurately reflect this mixed signal behavior. Acquired voxels are almost always interpolated to a finer in-plane resolution by scanner reconstruction and further smoothed in-plane by most image display software. As a result, the original acquired voxel structure is rarely visible in standard clinical images. Most commonly used mapping sequences acquire anisotropic voxels for two key reasons. First, while the in-plane voxel size typically ranges from 1.4–2 × 1.4–2 mm; the phase-encode direction often has a coarser resolution, sometimes exceeding 2 mm, to allow for shorter single-shot acquisition. Second, in two-dimensional imaging, the excited slice thickness for mapping methods is usually between 6 and 10 mm. As a result, thin-walled cardiac structures are not always well represented when the myocardial wall is not perpendicular to the imaging slice. Subendocardial or subepicardial voxels often contain a mixture of myocardium, blood, or fat, depending on their location (Fig. 8). Additionally, in conical structures such as the left ventricle, part of the voxel volume may include non-myocardial tissue, affecting pixel values in ways that are not readily apparent. Reducing partial volume effects can be achieved by increasing in-plane resolution—though this must be balanced against myocardial motion during image acquisition—reducing slice thickness and carefully aligning slices with the left ventricular walls. Near-isotropic and high-resolution voxel acquisition has been demonstrated using three-dimensional (3D) methods that register multiple stacks of T1 maps, though this requires extended scan times [65].

## 7 Magnetic susceptibility and off-resonance artifacts

### 7.1 Basic background

In MRI, the main magnetic field (B₀) aligns magnetic spins within an object, causing them to precess at a frequency proportional to the field strength. For most MRI techniques, a homogeneous field between applied gradient pulses is ideal to ensure uniform spin precession. However, materials with varying magnetic susceptibilities can locally distort the main magnetic field, leading to spatial variations in precession frequencies [66]. Most biological tissues exhibit diamagnetism, weakly opposing the magnetic field, whereas certain substances, including metal implants, enhance the field, creating spatially varying B₀ distributions [66]. This deviation from the expected resonance frequency is commonly referred to as off-resonance. Conventional inversion or saturation pulses used in T1 mapping techniques have a spectral bandwidth of approximately 1 kHz [67], [68]. Metal implants can shift the local resonance frequency by 2–6 kHz at distances of 5–10 cm, preventing spins outside the spectral bandwidth from being adequately inverted or saturated [68]. This disrupts the assumption of near-perfect inversion or saturation that underpins T1 mapping and can also cause spatial misregistration or signal cancellation. To counteract these effects, B₀ shimming is commonly applied to actively homogenize the magnetic field. However, this technique assumes smooth and gradual variations in B₀ and is often ineffective near metal implants or in regions with insufficient signal, such as the lungs.

### 7.2 Off-resonance due to susceptibility

**Fig. 9:**
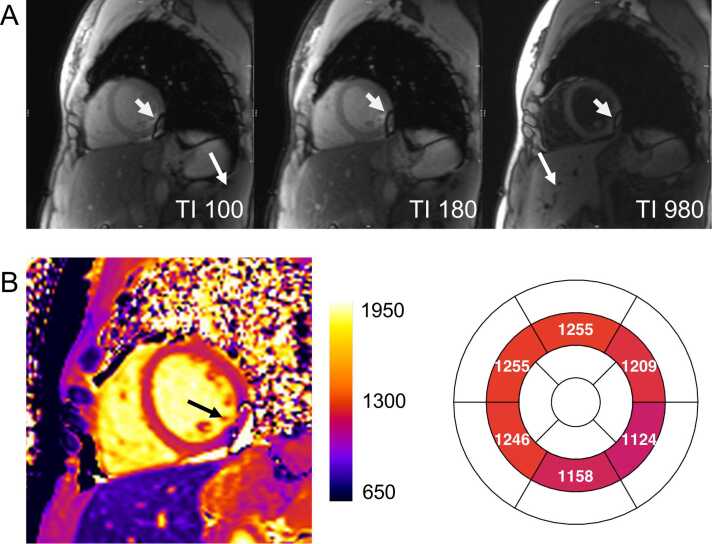
Off-resonance artifact caused by magnetic susceptibility differences between air-filled lungs and myocardium at 3T. (A) Motion-corrected source images demonstrate a focal inferolateral region of signal loss (white arrows), attributed to off-resonance effects resulting from magnetic susceptibility differences between the air-filled lungs and myocardium. (B) This artifact led to significantly reduced T1 values in the affected area and neighboring segments. These segments should be excluded from analysis, and the remaining segments interpreted with caution. *TI* inversion time

Susceptibility artifacts in cardiac imaging frequently occur in the myocardium adjacent to the lungs, leading to lower T1 values in areas with frequency offsets. While most biological tissues are diamagnetic, air is relatively paramagnetic (as it has near-zero magnetization), creating a sharp susceptibility difference at lung–myocardium interfaces [69]. This interaction distorts the magnetic field and introduces off-resonance effects that can be subtle or pronounced (Fig. 9). A prior study demonstrated that even in healthy volunteers, myocardial segments adjacent to the lung exhibit significantly different T1 values (>5%) compared to the septum, despite no visible artifacts [70]. Volume B₀ shimming over the heart can reduce off-resonance errors. However, because standard shimming methods only correct for quadratic (second-order) variations in B₀ across the magnet bore, they cannot fully correct for highly localized distortions around the heart [71]. The European Association of Cardiovascular Imaging–endorsed Society for Cardiovascular Magnetic Resonance (SCMR) consensus statement on T1, T2, T2*, and extracellular volume mapping underscores the importance of optimizing magnetic field homogeneity [15]. Volume-selective B₀ shimming focused on the heart is strongly recommended at 1.5T and considered essential at 3T to mitigate off-resonance effects. Additionally, B₁ (RF) volume shimming, despite its limited effectiveness, is recommended at 3T [15].

### 7.3 Off-resonance due to implantable devices

**Fig. 10:**
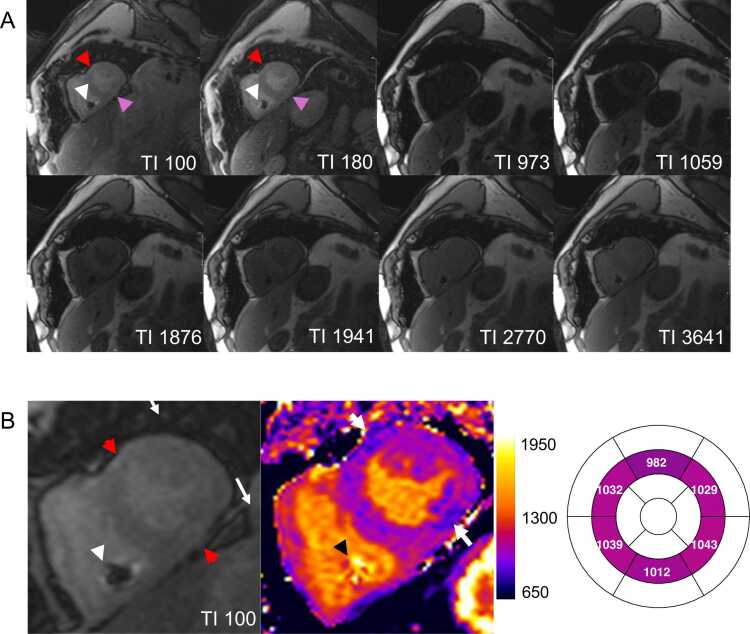
Off-resonance and susceptibility artifacts in a patient with a cardiac implanted electronic device, examined at 1.5T. (A) Motion-corrected source images show three artifacts: (1) off-resonance induced signal loss in the area of the right ventricular lead (A, white arrow). (2) Off-resonance in the anterior myocardial wall (red arrow) due to magnetic susceptibility differences between the air-filled lungs and myocardium. (3) Artifact in the inferior wall (pink arrow), most likely due to off-resonance effects. (B) The final map shows lower T1 values in these areas (enlarged first source image at TI = 100 ms provided for comparison) and should not be used for diagnosis making. *TI* inversion time

In cardiac MRI, patients frequently have metal implants such as cardiac implantable electronic devices (CIEDs), subcutaneous loop recorders, or sternal wires, all of which can induce severe off-resonance effects, leading to signal loss and banding artifacts. However, even without substantial artifacts or when there is no visual artifact, there might be an error in T1 in patients with CIEDs (as seen in Fig. 10). Bhuva et al. used off-resonance field mapping in patients with CIEDs and demonstrated that conventional MOLLI-based T1 measurements are unreliable in these patients, even in myocardial segments without apparent artifacts [72]. Additionally, metallic valve prostheses and sternal wires can cause localized field inhomogeneities, leading to errors in T1 and T2 measurements, particularly at higher field strengths [72], [73]. To address these challenges, specialized mapping sequences have been developed, including wideband inversion pulses, T1 off-resonance correction, and sGRE readout techniques, which can mitigate image artifacts and produce accurate myocardial T1 maps in patients with CIEDs [72], [74], [75]. However, these sequences are not yet widely available, may reduce signal-to-noise ratio, and require further clinical validation. Simple practical strategies can also help mitigate image artifacts from CIEDs: increasing the distance between the device and the heart, for example, by raising the ipsilateral arm during scanning. To enhance patient comfort, a gauze bandage or elastic band can help stabilize the arm [76], [77]. Acquiring images during inspiratory breath-holds can further displace the heart from the device. Additionally, right-sided generator implantation has been shown to significantly reduce the number of cardiac segments affected by artifacts [76]. As off-resonance and field inhomogeneity issues are more pronounced at higher field strengths, recent developments in low-field scanning techniques hold promise for improving T1 and T2 mapping reliability in patients with devices [78], [79]. A recently published SCMR expert consensus statement offers detailed guidance on safety measures and sequence optimization for CMR imaging in patients with CIEDs [77].

### 7.4 Off-resonance due to other objects

**Fig. 11:**
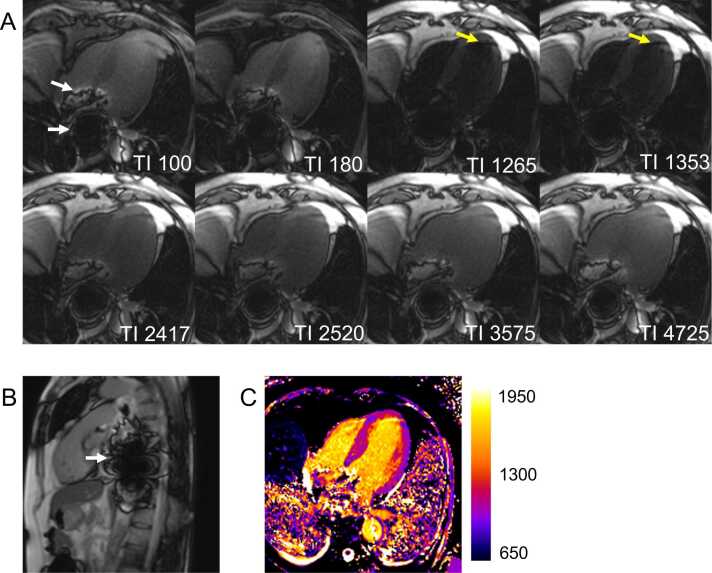
Off-resonance artifacts in a patient with an esophageal stent. (A) Source images reveal off-resonance induced signal loss in the region of the esophageal stent, accompanied by dark banding artifacts that extend to the basal ventricular myocardium (white arrows). An additional artifact band of varying signal impact is present across the apical heart (yellow arrows), which may reflect variable SSFP stabilization with varying inversion recovery time. (B) A sagittal localizer image further illustrates the extent of signal loss and dark banding artifacts. (C) While the left ventricular myocardium appears relatively unaffected by the artifacts on the final T1 map, the presence of the metal stent induces B₀ magnetic field inhomogeneities. These inhomogeneities likely result in errors in T1 estimates, particularly in myocardial regions closer to the banding artifacts (white arrows), compromising the diagnostic accuracy of the T1 map. The additional artifact, affecting the LV apex, can also be seen (black arrow). *SSFP* steady-state free precession, *TI* inversion time

Off-resonance artifacts in or near the heart can be caused by any material capable of locally altering the magnetic field. Fig. 11 demonstrates severe errors in T1 measurements induced by off-resonance due to an esophageal stent.

## 8 Gibbs ringing artifact

**Fig. 12:**
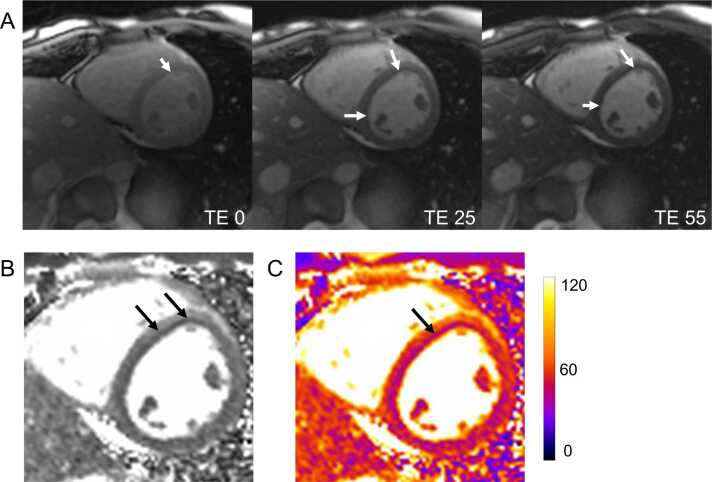
Gibb’s ringing. (A) T2-weighted source images, acquired in end-diastole showing a subendocardial septal dark band (white arrows), indicative of Gibbs ringing, which is also present on the final T2 map (B) and color-coded T2 map (C). Gibbs ringing commonly occurs at sharp interfaces between tissues with high differences in signal intensity, as observed here at the border between blood and myocardium. The dark band in myocardium is more prominent when the change in brightness at the blood and myocardium border is greater, which is typical for Gibbs ringing. Note that the Gibbs ringing is missing on the RV side of the septum which might be due to residual motion. *RV* right ventricular, *TE* echo time

Gibbs ringing in parametric mapping has been previously described [11], with an example shown in Fig. 12. This artifact arises from the abrupt truncation of high-frequency data in raw k-space sampling, mainly affecting pixels closest to sharp tissue borders [18]. Magnetic resonance images are reconstructed using Fourier transforms, which convert frequency-domain data into spatial representations. High-frequency components are essential for defining sharp edges and fine details. However, due to time constraints, as source images are typically acquired using single-shot techniques, only a limited region of k-space can be sampled. This incomplete representation of sharp edges results in oscillations around these structures, producing the visible artifact known as Gibbs ringing. The larger the pixels, the broader are the ripples (lower frequency) appearing deeper into the myocardium [18]. This artifact is more commonly observed along the phase-encoding direction. If the heart remains still during the single-shot acquisition, Gibbs ringing may become more pronounced. Additionally, it can sometimes be misinterpreted as a dark band caused by motion, as described earlier.

## 9 Wraparound

**Fig. 13:**
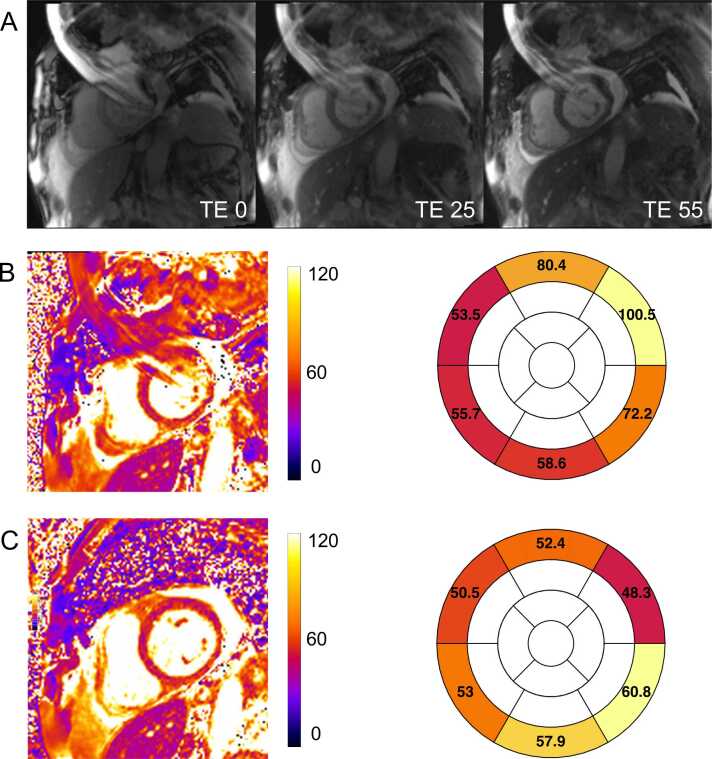
Infolding (wraparound) artifact in T2 mapping. (A) A wraparound artifact is visible on the source images and the final T2 map (B), causing significant artificial T2 elevation. Adjusting the field of view and changing the phase-encoding direction resolved the artifact, resulting in an accurate T2 map (C) and reliable T2 values. This example underscores the importance of thoroughly reviewing mapping images for artifacts, especially as AI-based contouring and batch processing are increasingly applied to large datasets. *AI* artificial intelligence, *TE* echo time

The phase-encode FOV wraparound artifact, or aliasing, occurs when the imaged anatomy exceeds the FOV in the phase-encoding direction [18]. This causes parts of the anatomy outside the FOV to appear at the opposite edge of the image. While techniques such as phase-oversampling can mitigate wraparound artifacts by expanding the FOV, they also increase the single-shot image duration, potentially affecting mapping values and amplifying cardiac motion artifacts. Thus, such techniques are not recommended for cardiac mapping procedures. To minimize wraparound, the phase-encoding direction is generally set along the shortest anatomical dimension. However, as long as the artifact does not reach the region of interest, accuracy and precision of mapping values remain unaffected. A typical wraparound artifact is shown in Fig. 13. This example underscores the importance of thoroughly reviewing mapping images for artifacts, especially as AI-based contouring and batch processing are increasingly applied to large datasets.

## 10 Lipomatous metaplasia

**Fig. 14:**
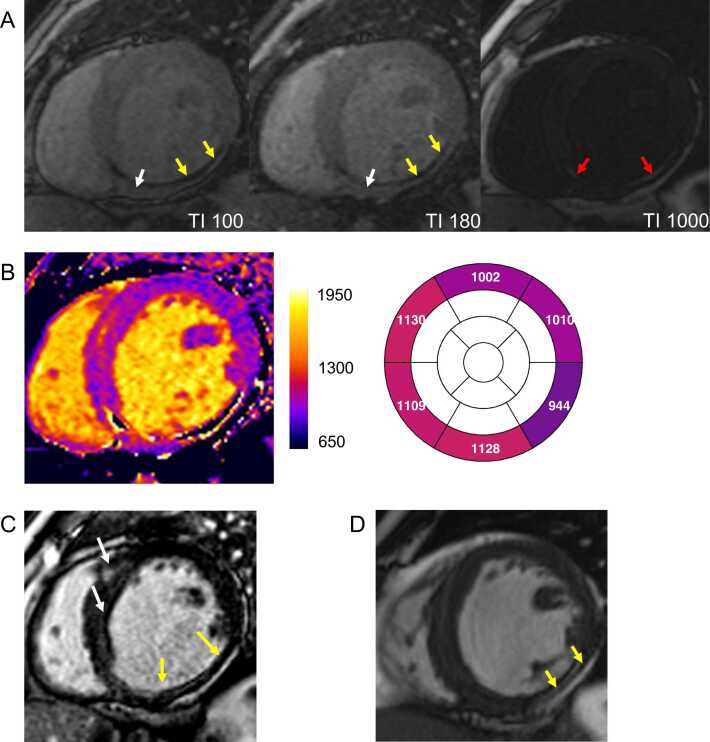
Lipomatous metaplasia in a patient with chronic myocardial infarction. (A) T1-weighted source images show the presence of different tissue types (white, yellow, and red arrows) in the basal inferior wall, reflected by different signal behavior following inversion. (B) Native T1 mapping reveals elevated T1 values in the basal septal and inferior segments (normal 2SD range 905–1037 ms), consistent with chronic myocardial infarction (C, yellow arrows), and focal non-ischemic fibrosis (C, white arrows) as demonstrated on LGE images. In contrast, the basal inferolateral segment shows low-normal T1 values despite evidence of chronic myocardial infarction on LGE images. This discrepancy is likely due to lipomatous metaplasia, characterized by shorter T1 values and visible as intramyocardial hyperintensity on bSSFP cine images (D, yellow arrows). It is crucial to differentiate this pathology from artifacts, as these mapping images provide valuable diagnostic information and should not be excluded from analysis. *bSSFP* balanced steady-state free precession, *LGE* late gadolinium enhancement, *TI* inversion time

Some pathologies can resemble artifacts in CMR images and lead to incorrect rejection of mapping images. An example is lipomatous metaplasia (LM), which can appear as a dark band on T1 maps (Fig. 14). LM refers to the appearance of mature white adipose tissue within infarcted myocardium [80]. Fat shortens T1 values and areas of myocardial lipid accumulation can appear as dark bands on color-coded T1 mapping images and are likely to be mistaken for artifacts (especially in the segment shown in Fig. 14). However, T1 signal characteristics of fat depend on sequence parameters, off-resonance effects, and field strength [81]. The combination of scar and fat can lead to either elevated or reduced T1 values depending on phase offset [81].

## 11 Discussion

T1 and T2 mapping are routinely used for myocardial tissue characterization and diagnosis of various myocardial diseases. Their reliability depends on awareness and understanding of artifacts and influencing factors. This review outlines common artifacts that may impair mapping accuracy—including those caused by motion, partial volume effects, PI inconsistencies, off-resonance, Gibbs ringing, and wraparound—and summarizes their typical sources and mitigation strategies.

Motion artifacts, often resulting from respiratory or cardiac motion, arrhythmias, or poor triggering, can lead to anatomical misalignment across source images. These can be reduced through breath-holding, imaging during the most quiescent cardiac phase (diastole or systole in arrhythmias), and application of non-rigid MOCO, although correction is more effective for respiratory than cardiac motion. Respiratory motion may also introduce PI artifacts, which can be mitigated by improved breath-hold instruction, simultaneous coil sensitivity information acquisition, or novel navigator- and pilot-tone–based free-breathing approaches. Partial volume artifacts arise when voxels include mixed tissue types, leading to signal averaging that violates mapping assumptions. They are especially problematic in thin myocardial regions or when using anisotropic voxels. Strategies include higher spatial resolution, reduced slice thickness, and, potentially, 3D isotropic acquisition. Off-resonance artifacts, caused by magnetic field inhomogeneities near air–tissue interfaces or metallic implants, may produce local T1/T2 errors even in the absence of visible image degradation. Mitigation includes targeted B₀ shimming, wideband or off-resonance-corrected sequences, modified patient positioning, and—potentially—low-field imaging. Additional artifacts, such as Gibbs ringing and wraparound, also affect image integrity. Gibbs artifacts occur at sharp tissue interfaces due to limited k-space sampling, while wraparound results from anatomy folding into the FOV. The latter can be addressed by altering phase-encoding direction, though not without trade-offs, or if necessary increasing the FOV, subject to possible effects on mapping results. This review does not aim to be exhaustive but focuses on common artifacts seen in our clinical practice. We acknowledge that artifact types, frequency, and mitigation strategies may differ across vendors and field strengths. As mapping techniques evolve—especially with free-breathing and 3D methods—future efforts should aim to classify artifacts more systematically and support automated quality control. We encourage the CMR community to expand this work by contributing examples and solutions beyond those covered here.

## Author contributions

**Jan Gröschel:** Writing – review & editing, Visualization. **Maximilian Fenski:** Writing – review & editing, Writing – original draft, Visualization, Validation, Project administration, Methodology, Conceptualization. **Christoph Kolbitsch:** Writing – review & editing, Visualization, Conceptualization. **Peter Gatehouse:** Writing – review & editing, Visualization, Methodology. **Jeanette Schulz-Menger:** Writing – review & editing, Supervision, Conceptualization.

## Declaration of competing interests

Maximilian Fenski receives funding from the 10.13039/501100005971German Heart Foundation.
